# Synthesis of Spherical Silver-coated Li_4_Ti_5_O_12_ Anode Material by a Sol-Gel-assisted Hydrothermal Method

**DOI:** 10.1186/s11671-017-2342-z

**Published:** 2017-10-30

**Authors:** Jun Li, Si Huang, Shuaijun Xu, Lifang Lan, Lu Lu, Shaofang Li

**Affiliations:** Faculty of Chemical Engineering and Light Industry,, Guangdong University of Technology, No. 100 Waihuan xi Road, Guangzhou Higher Education Mega Center, Panyu District, Guangzhou, Guangdong 510006 China

**Keywords:** Silver coating, Li_4_Ti_5_O_12_/Ag, Lithium-ion batteries, Electrochemical performance

## Abstract

**ᅟ:**

Ag-coated spherical Li_4_Ti_5_O_12_ composite was successfully synthesized via a sol-gel-assisted hydrothermal method using an ethylene glycol and silver nitrate mixture as the precursor, and the influence of the Ag coating contents on the electrochemical properties of its was extensively investigated. X-ray diffraction (XRD) analysis indicated that the Ag coating does not change the spinel structure of Li_4_Ti_5_O_12_. The electrochemical impedance spectroscopy (EIS) analyses demonstrated that the excellent electrical conductivity of the Li_4_Ti_5_O_12_/Ag resulted from the presence of the highly conducting silver coating layer. Additionally, the nano-thick silver layer, which was uniformly coated on the particles, significantly improved this material’s rate capability. As a consequence, the silver-coated micron-sized spherial Li_4_Ti_5_O_12_ exhibited excellent electrochemical performance. Thus, with an appropriate silver content of 5 wt.%, the Li_4_Ti_5_O_12_/Ag delivered the highest capacity of 186.34 mAh g^−1^ at 0.5C, which is higher than that of other samples, and maintained 92.69% of its initial capacity at 5C after 100 cycles. Even at 10C after 100 cycles, it still had a capacity retention of 89.17%, demonstrating remarkable cycling stability.

**Trial registration:**

ISRCTN NARL-D-17-00568

## Highlights

1. Spherical Li_4_Ti_5_O_12_/Ag composites were synthesized via a sol-gel-assisted hydrothermal method using ethylene glycol and a silver nitrate mixture as the precursor for the coating layer, which significantly improved the electronic conductivity and the electrochemical performance of Li_4_Ti_5_O_12_.
2. The spherical morphology could induce a large tap density and consequently enhance the volumetric energy density.

## Background

Over the last decade, rechargeable lithium-ion batteries (LIBs) have demonstrated many advantages. They are light weight; have a small size, high voltage and high energy density; and have been attracting intense interest as an electrochemical energy storage device to reduce exhaust emissions and for fuel economy [1, 2]. However, the price of lithium precursors, safety and life issues, and low power density are obstacles for the application of LIBs for large-scale energy storage in the future [3]. Therefore, to develop substitute materials to meet the demands of the safety of the large-scale storage, great efforts have been made [4].

Cube spinel lithium titanate (Li_4_Ti_5_O_12_) materials, the anode materials of Li-ion batteries, have been become a promising material because of their zero-strain structural characteristic during the intercalation and deintercalation process of Li_4_Ti_5_O_12_ [5–9]. This material has a platform lithium insertion and extraction voltage of ~ 1.55 V (vs. Li/Li^+^), avoiding the formation of lithium-consuming solid electrolyte interface (SEI) films, which should be beneficial for enhancing safety and good cycling of the LIBs. Therefore, Li_4_Ti_5_O_12_ has become one of the potential materials in commercial applications and scientific research. Li_4_Ti_5_O_12_ has been prepared via various of methods, for example, solid-state, electroless deposition, microwave, and sol-gel method. Regarding the solid-state method, some studies have shown that it has a simple synthesis route and low synthesis cost because of the shorter distance for Li^+^ diffusion and electron transfer, Li_4_Ti_5_O_12_ exhibits an excellent rate capability, but the solid-state reaction cannot provide a uniform morphology with a narrow size. However, the electroless deposition process has a complex synthesis route. For the sol-gel synthesis of Li_4_Ti_5_O_12_, several researchers have reported that it can yield products with a uniformly homogeneous distribution and narrow particles with good stoichiometric control.

Despite these many advantages, the major drawbacks of Li_4_Ti_5_O_12_ are its poor electronic and ionic conductivity and its slow Li-ion diffusion coefficient, which results in a poor rate capacity. Numerous strategies, including crystallite size reduction [10], doping with high valence metal ions [11–13], and coating with conducting phases [14–17], have been adopted to improve the discharge/charge transport properties of electrodes. In addition, another way to enhance the electronic conductivity is to synthesize nanostructured Li_4_Ti_5_O_12_. The nanostructures provide a larger electrode/electrolyte contact area to increase the intercalation kinetics and reduce the diffusion paths to accelerate Li^+^ and electron transport [18]. Among these approaches, the most effective way to improve the electrochemical properties of Li_4_Ti_5_O_12_ is conductive surface modification. Aslihan et al. [2] synthesized Li_4_Ti_5_O_12_ via the sol-gel method, and then the as-synthesized Li_4_Ti_5_O_12_ was surface-coated with silver via electroless deposition. The results showed that silver coating (Ag coating) affords a highly conductive matrix for Li^+^ insertion, improving the electronic conductivity. Zhu et al. [19] prepared carbon-coated nano-sized Li_4_Ti_5_O_12_ nanoporous micro-spheres with a remarkable rate capability via a carbon pre-coating process in combination with a spray drying method, and indicated that the micron-sized spherical particles induce a large tap density, resulting in the enhancement of the volumetric energy density. However, how to synthesize Ag-coated micron-sized Li_4_Ti_5_O_12_ spherical particles via a sol-gel-assisted hydrothermal method has not been reported.

Herein, we report a sol-gel-assisted hydrothermal method to synthesize the micron-sized spherical Li_4_Ti_5_O_12_/Ag composite using ethylene glycol and a silver nitrate mixture as a precursor, and the content of the Ag coating was adjusted by controlling the amount of silver element in the precursor. The electrochemical properties of the Li_4_Ti_5_O_12_/Ag with spherical morphology were investigated in detail.

## Experimental

### Synthesis of Pristine Li_4_Ti_5_O_12_ and Modification of its Surface with Ag

#### Synthesis of the Spherical Precursor Via the Sol-Gel Method

The spherical precursor titanium glycolate (TG) was synthesized by the sol-gel method. First, 2 mL of tetrabutyl titanate was added slowly to the solution, which contained AgNO_3_ (at an appropriate amount to be soluble in 50 mL of glycol), under vigorous stirring to form the precursor solution. Second, the precursor solution was added to a 150 mL of acetone mixture that contained 0.1 mL Tween 80, and agitation was continued for 1 h at room temperature to form precipitates. Then, the precipitates were aged for 8 h, separated by filtration, and washed twice with anhydrous alcohol. Finally, the precursor powders were obtained by heat treatment at 80 °C for 6 h in an oven followed by grinding.

#### Synthesis of Spherical Li_4_Ti_5_O_12_/Ag

The spherical Li_4_Ti_5_O_12_/Ag was prepared via the hydrothermal method. First, LiOH·H_2_O and the precursor in a molar ratio of 3.9:1 were homogeneously mixed by stirring with 40 mL of alcohol as the media for 1 h to form a mixture, which was then heated at 180 °C for 12 h in sealed Teflon wares until precipitates were obtained. Second, the precipitates were collected via centrifugation (5000 rpm, 5 min) and further washed with anhydrous ethanol several times. Then, they were dried in an oven at 80 °C for 2 h. Finally, the precipitates were heated in a muffle furnace at 700 °C for 2 h (heating rate of 5 °C·min^−1^) in air after they were ground and then naturally cooled to room temperature to obtain spherical Li_4_Ti_5_O_12_/Ag powder.

### Material Characterization

The structure of the Li_4_Ti_5_O_12_ samples were identified via X-ray diffraction (XRD, Rigaku D/max-PC2200) using a Cu Kα radiation (λ = 0.15405 nm) source with a scan rate of 4 °min^−1^ from 10° to 80° and operated at 40 KV and 20 mA. The morphology and particle size of the materials were explored via SEM (scanning electron microscopy, Supra 55 Zeiss) and TEM (transmission electron microscopy, JEOL-2100).

### Electrochemical Measurements

The electrochemical performances of the products were tested using a CR2025 coin-type cell. The working electrodes were prepared by mixing 80 wt.% Li_4_Ti_5_O_12_/Ag active materials, 10 wt.% conductive Super-P, and 10 wt.% polyvinylidene fluoride (PVDF) binder in N-methyl-2-pyrrolidone (NMP) solvent to form a uniform slurry. Then, the slurry was cast onto an aluminum foil and dried under vacuum at 80 °C for 12 h to remove the residual solvent. Then, the foil was pressed and cut into disks. A Celgard 2400 polypropylene microporous membrane and lithium foil were used as the separator and the negative electrode, respectively. The electrolyte solution was 1 M LiPF_6_ in ethylene carbonate (EC), dimethyl carbonate (DMC), and ethylene methyl carbonate (EMC) in a volumetric ratio of 1:1:1. The cells were assembled in an argon-filled glove box, where both the moisture and oxygen levels were kept below 1 ppm. The electrochemical tests of the products were evaluated using a LAND CT2001A test system (Wuhan, China). Cyclic voltammetry (CV) tests were recorded on a CHI600A electrochemical workstation at a 0.1 mV s^−1^ scan rate from 1.0 to 2.5 V (vs. Li/ Li^+^). EIS measurements were performed in the frequency range of 100 KHz to 10 mHz with a perturbation of 5 mV.

## Results and Discussion

### Structural and Morphological Properties

**Fig. 1:**
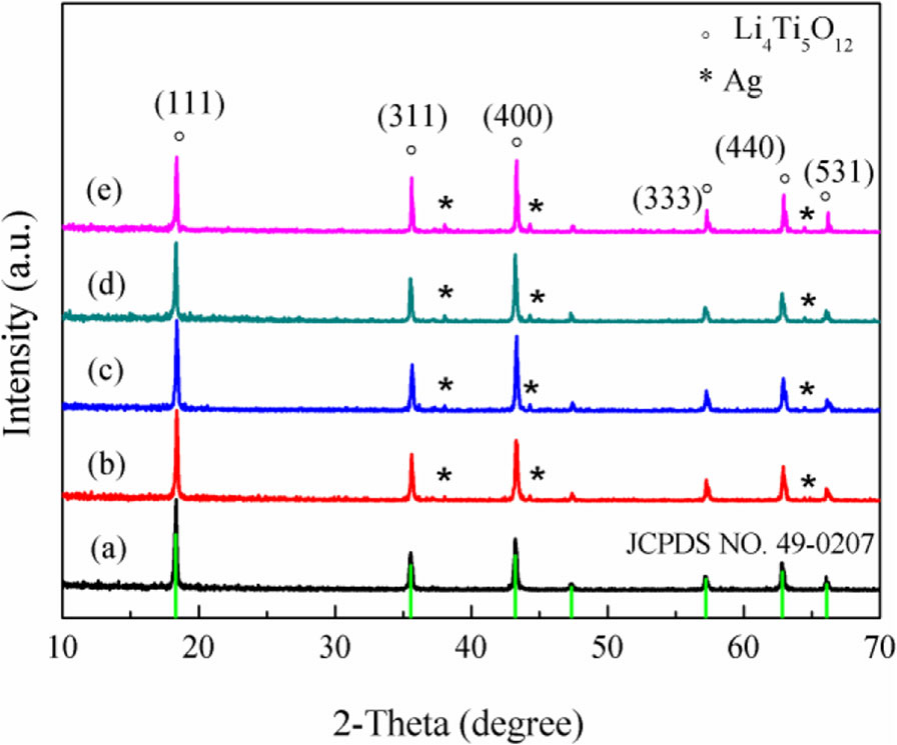
XRD patterns of the Li_4_Ti_5_O_12_/Ag. (**a**) 0 wt.%, (**b**) 1 wt.%, (**c**) 3 wt.%, (**d**) 5 wt.%, and (**e**) 7 wt.%

The effect of the amount of Ag additive on the Li_4_Ti_5_O_12_/Ag powders was investigated. The XRD patterns of the Ag-coated spherical Li_4_Ti_5_O_12_ composites are given in Fig. 1. It can be easily seen that the major diffraction peaks of all specimens appear at 18.4°, 35.54°, 43.2°, 57.2°, 62.8°, and 66.1° and are indexed as the (111), (311), (400), (333), (440), and (531), respectively. That peaks are in good agreement with the Li_4_Ti_5_O_12_ standard diffraction pattern [20], except for characteristic patterns of the Ag metal (2θ = 38.1°, 44.3°, 64.4°). No impurity diffraction peaks were detected in any of the specimens. Moreover, the peak intensity of silver correspondingly increased as the amount of Ag increased.

Lattice parameters of the Li_4_Ti_5_O_12_/Ag samples with different Ag coatings are provided in Table 1. No significant changes with the Ag content increase were observed. Thus, it was suggested that silver is mainly coating in the form of the elemental Ag on the surface of Li_4_Ti_5_O_12_ particles but not penetrating into the lattice of spinel Li_4_Ti_5_O_12_. Because the ionic radius of Ag^+^ (0.126 nm) is substantially larger than that of the Ti^4+^ (0.068 nm), the as-synthesized Li_4_Ti_5_O_12_/Ag sample was just a composite of the Ag metal and the Li_4_Ti_5_O_12_ phase.

**Table 1:** Lattice parameters of the Li_4_Ti_5_O_12_/Ag composites coated with different Ag contents

Content of Ag, wt.%	Lattice parameter (a = b = c), nm
0	0.83577
1	0.83572
3	0.83574
5	0.83576
7	0.83578

**Fig. 2:**
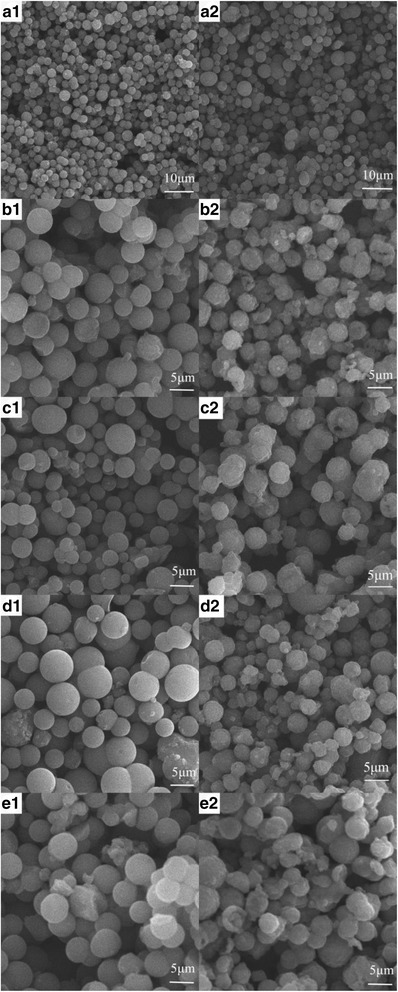
SEM images of the precursor and the Li_4_Ti_5_O_12_/Ag. (**a**) 0 wt.%, (**b**) 1 wt.%, (**c**) 3 wt.%, (**d**) 5 wt.%, (**e**) 7 wt.%

Figure 2 shows the SEM images of the as-prepared precursor (a_1_-e_1_) and Li_4_Ti_5_O_12_/Ag (a_2_-e_2_). As shown in Fig. 2, all samples exhibit a uniformly spherical structure with a narrow size distribution of 5–10 μm, which is beneficial to a contact between the active materials and electrode. From the SEM images, the spherical precursor, titanium glycolate (TG) particles, shows a smooth line, whereas the Li_4_Ti_5_O_12_/Ag particles presents a rough line. Moreover, a good dispersion could enlarge the electrode-electrolyte contact area and significantly accelerate the transportation of Li^+^ and electron. However, the surface of the Li_4_Ti_5_O_12_/Ag samples are not obviously smoother than that of the as-prepared precursor and titanium glycolate, and they exist to a certain extent as an agglomeration. Moreover, the particle sizes of different Li_4_Ti_5_O_12_/Ag composites are much larger than that of Ag-free Li_4_Ti_5_O_12_; however, the agglomeration phenomenon becomes more obvious with an increasing in silver content.

**Fig. 3:**
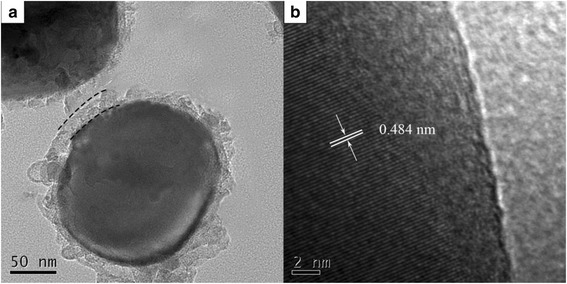
(**a**) TEM and (**b**) HRTEM images of the 5 wt.% silver-coated Li_4_Ti_5_O_12_, in which the “line” indicates the coated silver layer

The distribution of silver in the interior of the micron-sized particles was further investigated, and TEM and HRTEM analyses were provided in Fig. 3. The TEM images (Fig. 3a) show that the 5 wt.% Ag-coated micron-sized-spherical Li_4_Ti_5_O_12_ particles are uniformly coated by a silver layer with a thickness of 3~4 nm, indicating that the silver layer builds a conductive network on the surface of the entire material, which facilitates the lithium ion and electron transport. As shown in Fig. 3b, the surface of the micron-sized Li_4_Ti_5_O_12_/Ag particles are not smooth, and the *d*-spacing of the 5 wt.% Ag-coated Li_4_Ti_5_O_12_ particles is 0.484 nm, which matches well with that of the LTO (111) plane. This suggests that no new phase was generated on the surface of the LTO particles, but there was a thin coating layer on the particles.

### Electrochemical Properties

**Fig. 4:**
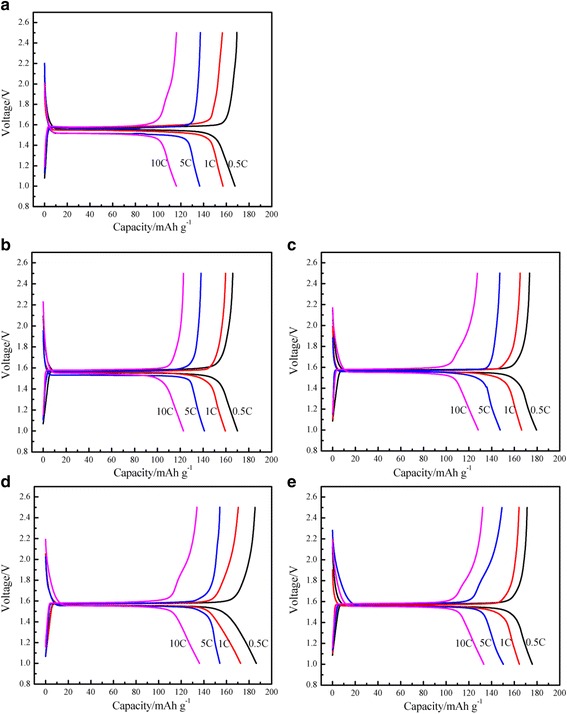
The initial charge-discharge curves with various current densities of the Li_4_Ti_5_O_12_/Ag. (**a**) 0 wt.%, (**b**) 1 wt.%, (**c**) 3 wt.%, (**d**) 5 wt.%, (**e**) 7 wt.%

Figure 4 shows the first charge-discharge curves of the micron-sized spherical Li_4_Ti_5_O_12_/Ag electrodes coated with different Ag contents at the different rates. As it can be seen from Fig. 4, all of the profiles exhibit an extremely flat voltage plateau of 1.55 V (vs. Li/Li^+^), indicating a two-phase transition between Li_4_Ti_5_O_12_ and Li_7_Ti_5_O_12_ for lithium insertion [21]. The voltage platform of Li_4_Ti_5_O_12_/Ag composites is longer than that of Ag-free Li_4_Ti_5_O_12_. With an increasing content of Ag, for a longer discharge platform of the Li_4_Ti_5_O_12_/Ag composites, the ability to maintain the platform is stronger, suggesting that good electronic conductivity can effectively reduce the polarization of the material during the electrode reaction process, and improve the utilization of the material.

As shown in Fig. 4, Ag-free Li_4_Ti_5_O_12_ delivered an initial discharge-specific capacity of 167.62 mAh g^−1^ at a rate of 0.5C, whereas the delivered capacity of the Ag-coated micron-sized spherical Li_4_Ti_5_O_12_ composites increased with increasing silver amount: 170.10, 179.54, and 186.34 mAh g^−1^ for 1, 3, and 5 wt.%, respectively. But 7 wt.% Ag-coated Li_4_Ti_5_O_12_ exhibited a somewhat different behavior. The delivered discharge-specific capacity decreased with increasing silver amount: 175.86 mAh g^−1^ for 7 wt.%. The 5 wt.% Ag-coated Li_4_Ti_5_O_12_ gained the highest initial discharge capacity, and the initial discharge-specific capacities reached 186.34, 172.47, 154.12, and 136.06 mAh g^−1^ at the specific currents of 0.5, 1, 5, and 10C, respectively. Due to the poor electronic conductivity and sluggish Li^+^ diffusion, the material exhibits a large polarization at high charge/discharge rates. The highly conductive Ag additive can significantly enhance the surface intercalation reaction and reduce the polarization [20, 22]. Even the highest Ag content (7 wt.%) can provide the longest voltage plateau, and the metal silver itself cannot be fully intercalated into the lithium. Instead, the high content of Ag will lead to a decrease in the specific capacity of Li_4_Ti_5_O_12_/Ag. Therefore, an appropriate silver content can not only effectively improve the conductivity of the Li_4_Ti_5_O_12_ and reduce the polarization of the Li_4_Ti_5_O_12_ in the reaction process but can also reduce the loss of the reversible capacity due to the Ag coating.

**Fig. 5:**
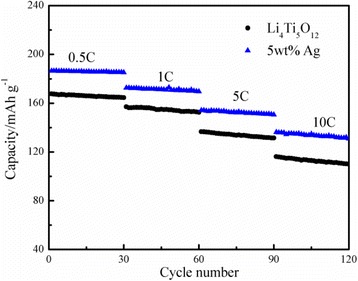
Rate capability of the Ag-free and the 5 wt.% Ag-coated Li_4_Ti_5_O_12_ under different current rates

**Fig. 6:**
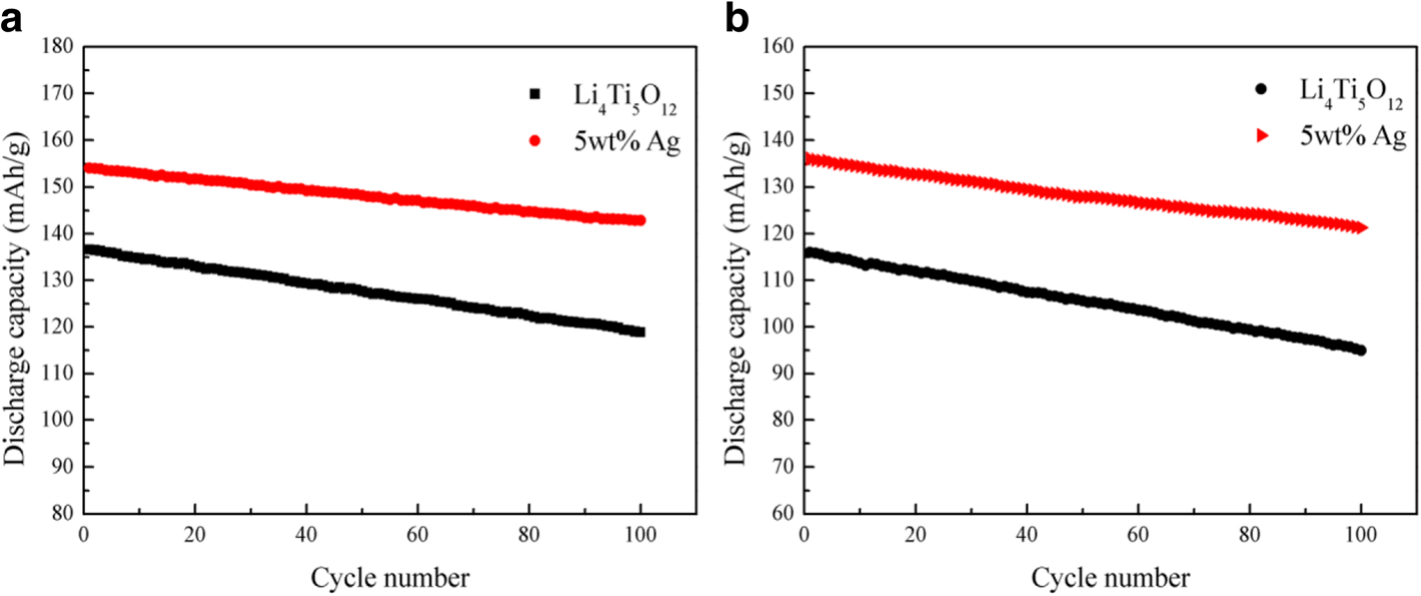
The cycling performance of the Ag-free and the 5 wt.% Ag-coated Li_4_Ti_5_O_12_ at 5 °C (**a**) and 10 °C (**b**)

The rate capabilities of the Ag-free Li_4_Ti_5_O_12_ and 5 wt.% Ag-coated Li_4_Ti_5_O_12_ composite were analyzed at current densities of 0.5, 1, 5, and 10C, and the results are shown in Fig. 5. As shown, the initial capacity of the 5 wt.% Ag-coated Li_4_Ti_5_O_12_ composite at 5C was 154.12 mAh g^−1^. After 30 cycles, the capacity was still maintained at 150.50 mAh g^−1^, retaining over 97.65% of the initial capacity. When it was further increased to 10C, the discharge capacity apparently dropped from 136.06 mAh g^−1^ to 130.81 mAh g^−1^ after 30 cycles. While the retention efficiency of the capacity could still be maintained at 96.14%. What is more, the cycling performance of the Li_4_Ti_5_O_12_/Ag composite was significantly better than that of the Ag-free Li_4_Ti_5_O_12_ at various charge-discharge rates. As shown in Fig. 6a, with an appropriate silver contents of 5 wt.%, the silver-coated Li_4_Ti_5_O_12_ delivered the highest capacity of 186.34 mAh g^−1^ at 0.5C, which is higher than that of other samples, and maintained 92.69% of its initial capacity at 5C after 100 cycles. Even at 10C after 100 cycles (Fig. 6b), it still had a capacity retention of 89.17%, demonstrating remarkable cycling stability. The results suggested that under the favorable experimental conditions, the Li_4_Ti_5_O_12_ surface Ag coating not only enhanced the electron and ionic conductivity but also obviously increased the electron transport during the lithium insertion/extraction reaction and significantly improved the cycle stability of the Li_4_Ti_5_O_12_.

**Fig. 7:**
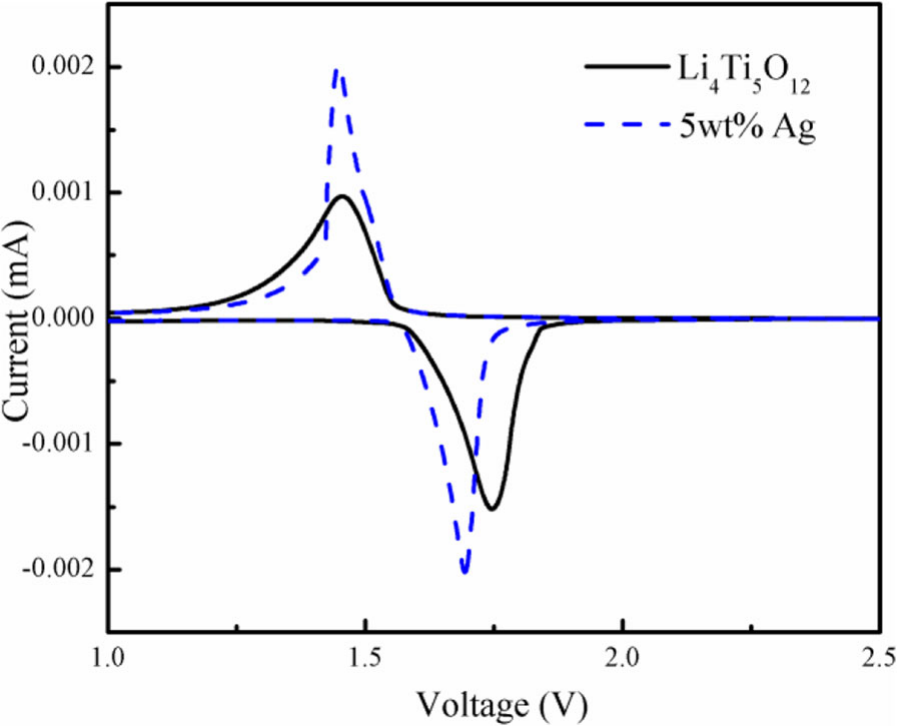
CV curves of the Ag-free and the 5 wt.% Ag-coated Li_4_Ti_5_O_12_ composite

Figure 7 presents the cyclic voltammograms (CVs) of the Ag-free Li_4_Ti_5_O_12_ and 5 wt.% Ag-coated Li_4_Ti_5_O_12_ composite obtained at a slow rate of 0.1 mV s^−1^. Obviously, reversible redox peaks between 1.0 and 2.5 V were obtained, which are attributed to the insertion and extraction of lithium ions, suggesting no intermediate phase formation during lithium insertion and de-insertion. Meanwhile, the redox peak area of these two curves is almost equal, indicating a high coulombic efficiency [23]. The potential differences between the oxidation and reduction peaks of the 5 wt.% Ag-coated Li_4_Ti_5_O_12_ is 0.244 V, which is slightly lower than that of the Ag-free Li_4_Ti_5_O_12_ (0.24 V). This suggests that appropriately surface coating the highly conductive Ag additive significantly reduced the polarization of the Li_4_Ti_5_O_12_ sample and effectively improved its electrochemical performance. Moreover, the redox peaks of the 5 wt.% Ag-coated Li_4_Ti_5_O_12_ are sharper and larger than that of Ag-free Li_4_Ti_5_O_12_, which indicates that an appropriate Ag coating can improve the dynamic performance of the electrode.

**Fig. 8:**
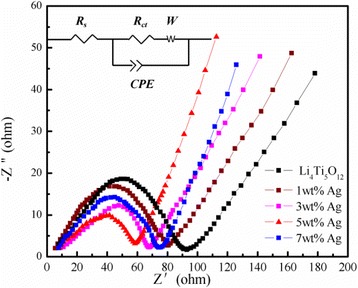
EIS patterns of the pure Li_4_Ti_5_O_12_ and the Li_4_Ti_5_O_12_ coated with different Ag contents

Electrochemical impedance spectroscopy (EIS) measurements of Ag-free Li_4_Ti_5_O_12_ and 5 wt.% Ag-coated Li_4_Ti_5_O_12_ were conducted in the frequency range of 10^5^ to 0.01 Hz before galvanostatic cycles. Additionally, the equivalent circuit (inset) and corresponding impedance data are shown in Fig. 8. In the equivalent circuit, *Rs* represents the electrolyte solution resistance, which reflects the electric conductivity of the electrolyte, separator, and electrodes. (intersection with the *Z* ^′^ axis at a high frequency), *R*ct shows the charge-transfer resistance in materials, *CPE* is the double-layer and passivation film capacitance, and *W* is the Warburg impedance, which is related to lithium ion diffusion in the low frequency region. The parameters obtained by fitting are listed in Table 2. As shown in Fig. 8, both EIS curves were composed of a depressed semicircle in the high-frequency region and an oblique straight line in the low-frequency region. The diameter of the semicircle stands for the charge-transfer resistance, and the oblique straight line is related to the Warburg impedance [24]. The impedance of the semicircles in the high frequency region correspond to the electrode and liquid electrolyte interface charge transfer process, and the straight line in the low frequency region can be expressed as the lithium ions’ diffusion behavior in the oxide structure [25–28]. As shown from Fig. 8, the diameter of the semicircle of the 5 wt.% Ag-coated Li_4_Ti_5_O_12_ is shorter than that of bare Li_4_Ti_5_O_12_, indicating that a proper amount of Ag coating could enhance the electronic conductivity of Li_4_Ti_5_O_12_, and this has to do with the charge-transfer process, where Li^+^ and electrons reach the electrode surface simultaneously to complete the reaction. This mainly depends on the redox reaction across the surface of the active materials. The smaller charge-transfer resistance of the 5 wt.% Ag-coated Li_4_Ti_5_O_12_ reflected a faster charge transfer reactions at their electrode/electrolyte interfaces.

**Table 2:** Impedance parameters of the pure Li_4_Ti_5_O_12_ and the Li_4_Ti_5_O_12_/Ag composites coated with different silver contents

Samples	R*s*/Ω	Rct/Ω	D_Li_/cm^2^ s^−1^
Li_4_Ti_5_O_12_	5.84	87.16	8.69 × 10^−12^
Li_4_Ti_5_O_12_/Ag (1 mass%)	4.56	77.44	2.14 × 10^−11^
Li_4_Ti_5_O_12_/Ag (3 mass%)	4.38	66.62	3.75 × 10^−11^
Li_4_Ti_5_O_12_/Ag (5 mass%)	4.13	57.87	6.73 × 10^−11^
Li_4_Ti_5_O_12_/Ag (7 mass%)	4.47	70.53	4.69 × 10^−11^

The lithium ion chemical diffusion coefficient can be calculated from the plot in the low-frequency region by using the following Eq. (1) [29–33].

1 $$ {D}_{{\mathrm{Li}}^{+}}=\frac{R^2{T}^2}{2{A}^2{n}^4{F}^4{C}_{Li}^2{\sigma_w}^2} $$

Here, ${D}_{{\mathrm{Li}}^{+}}$ is the lithium-ion diffusion coefficient, *R* is the gas constant (8.314 JK mol^−1^), *T* is the absolute temperature (298 K), *A* is the surface area of the electrode, *n* is the number of electrons per molecule attending the electronic transfer reaction, *F* is the Faraday constant (96,500 C mol^−1^), *C*_Li_ is the concentration of lithium ions in the Li_4_Ti_5_O_12_ electrode, and *σ*_*w*_ is the Warburg factor, which has the following relationship with *Ζ*_re_:

2 $$ {Z}_{\mathrm{re}}={R}_S+{R}_{\mathrm{ct}}+{\sigma}_w\cdot {\omega}^{- 0.5} $$

**Fig. 9:**
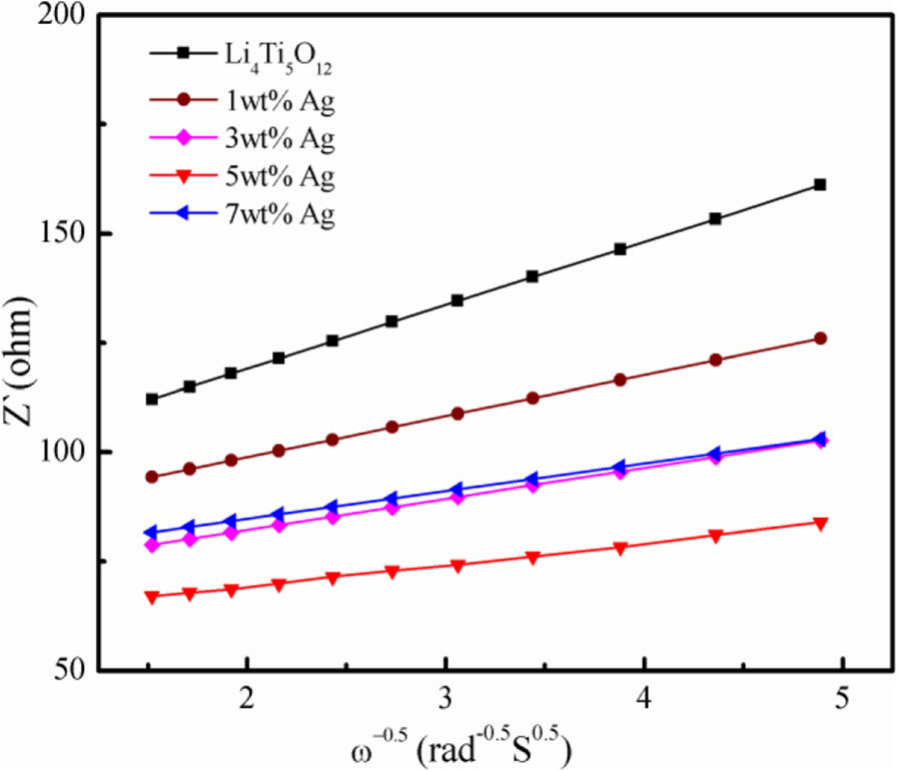
Graph of *Z*_re_ plotted against *ω* ^−0.5^ at the low frequency region for the Li_4_Ti_5_O_12_ and Li_4_Ti_5_O_12_ coated with different Ag contents

Additionally, the relationship between *Z*_re_ and the reciprocal square root of frequency in the low frequency is shown in Fig. 9. All of the parameters obtained and calculated from the EIS are summarized in Table 2. As shown in Table 2, ${D}_{\mathrm{L}{\mathrm{i}}^{+}}$ of the 5 wt.% Ag-coated Li_4_Ti_5_O_12_ is 6.73 × 10^−11^, which is one order of magnitude higher than that of Li_4_Ti_5_O_12_ (8.69 × 10^−12^). The 5 wt.% Ag-coated Li_4_Ti_5_O_12_ has the largest lithium diffusion coefficient compared with that of Ag-free Li_4_Ti_5_O_12_ and 1, 3, and 7 wt.% Ag-coated Li_4_Ti_5_O_12_ composites, indicating that coating with Ag is an effective way to improve the electronic conductivity. Consequently, the rate capacity of the 5 wt.% Ag-coated Li_4_Ti_5_O_12_ can be substantially improved.

## Conclusions

Anode materials spherical Li_4_Ti_5_O_12_/Ag composites with a high tap density were prepared by a sol-gel-assisted hydrothermal method. The electrochemical tests show that the appropriate amount of Ag coating can significantly improve the electronic conductivity of Li_4_Ti_5_O_12_ and enhance the cycle stability. The optimum content of silver is 5wt.%, which can get excellent electrochemical performance. However, the excessive silver content will cause the electrochemical properties of material worse. Therefore, appropriate Ag-coated spherical Li_4_Ti_5_O_12_ composite is a superior lithium storage material with a high capacity and excellent safety, and it has real potential as a promising material in power lithium ion batteries.
